# CT-based Assessment at 6-Month Follow-up of COVID-19 Pneumonia patients in China

**DOI:** 10.1038/s41598-024-54920-1

**Published:** 2024-02-29

**Authors:** Xingyu Fang, Yuan Lv, Wei Lv, Lin Liu, Yun Feng, Li Liu, Feng Pan, Yijun Zhang

**Affiliations:** 1 Department of Radiology, the 305 Hospital of PLA, 13 Wenjin Street, Beijing, 100017 China; 2 Medical Department of General Surgery, Chinese PLA General Hospital, The 1St Medical Center, Beijing, 100853 China; 3 Department of General Surgery, The 7Th Medical Center, Chinese PLA General Hospital, Beijing, 100700 China

**Keywords:** COVID-19, SARS-CoV-2, Pneumonia, Tomography, X-ray, Follow-up, Viral infection, Risk factors, Respiratory tract diseases

## Abstract

This study aimed to assess pulmonary changes at 6-month follow-up CT and predictors of pulmonary residual abnormalities and fibrotic-like changes in COVID-19 pneumonia patients in China following relaxation of COVID restrictions in 2022. A total of 271 hospitalized patients with COVID-19 pneumonia admitted between November 29, 2022 and February 10, 2023 were prospectively evaluated at 6 months. CT characteristics and Chest CT scores of pulmonary abnormalities were compared between the initial and the 6-month CT. The association of demographic and clinical factors with CT residual abnormalities or fibrotic-like changes were assessed using logistic regression. Follow-up CT scans were obtained at a median of 177 days (IQR, 170–185 days) after hospital admission. Pulmonary residual abnormalities and fibrotic-like changes were found in 98 (36.2%) and 39 (14.4%) participants. In multivariable analysis of pulmonary residual abnormalities and fibrotic-like changes, the top three predictive factors were invasive ventilation (OR 13.6; 95% CI 1.9, 45; P < .001), age > 60 years (OR 9.1; 95% CI 2.3, 39; P = .01), paxlovid (OR 0.11; 95% CI 0.04, 0.48; P = .01) and invasive ventilation (OR 10.3; 95% CI 2.9, 33; P = .002), paxlovid (OR 0.1; 95% CI 0.03, 0.48; P = .01), smoker (OR 9.9; 95% CI 2.4, 31; P = .01), respectively. The 6-month follow-up CT of recent COVID-19 pneumonia cases in China showed a considerable proportion of the patients with pulmonary residual abnormalities and fibrotic-like changes. Antivirals against SARS-CoV-2 like paxlovid may be beneficial for long-term regression of COVID-19 pneumonia.

## Introduction

Coronavirus disease 2019 (COVID-19), caused by severe acute respiratory syndrome coronavirus type 2 (SARS-CoV-2), has become a global pandemic for more than three years. COVID-19 has been proven to cause multi-organ damage, and pneumonia is the most common manifestation^1,2^. Chest CT plays an important role in the diagnosis, follow-up and presumed prognosis of patients with COVID-19^3,4^. Several studies have demonstrated permanent radiographic changes and possibility of pulmonary fibrosis during the follow-up CTs of COVID-19 patients^5–7^.

Since the beginning of the pandemic, SARS-CoV-2 has been evolving rapidly through genetic mutations during virus replication^8,9^. Although much evidence suggests that the pathogenicity of the virus is gradually diminishing as it evolves, the trend is not definitive^10,11^. Therefore, the clinical characteristics and long-term follow-up of COVID-19 still deserve continued attention.

China has experienced an outbreak of infections since the gradual deregulation of COVID-19 in the year-end of 2022. Beijing was one of the first and most severe outbreak areas, and the predominant SARS-CoV-2 variants at that time was Omicron BF.7 and BF.5.2, which had many new epidemiological and clinical characteristics^12,13^. This study aimed to assess pulmonary changes at 6-month follow-up CT and predictors of pulmonary residual abnormalities and fibrotic-like changes in recent COVID-19 pneumonia patients.

## Materials and methods

### Participants

This prospective study was approved by the Institutional Review Board of the 305 Hospital of PLA and the 7th Medical Center of Chinese PLA General Hospital. Informed consent was provided by all participants and the study was conducted in accordance with the Declaration of Helsinki.

**Figure 1:**
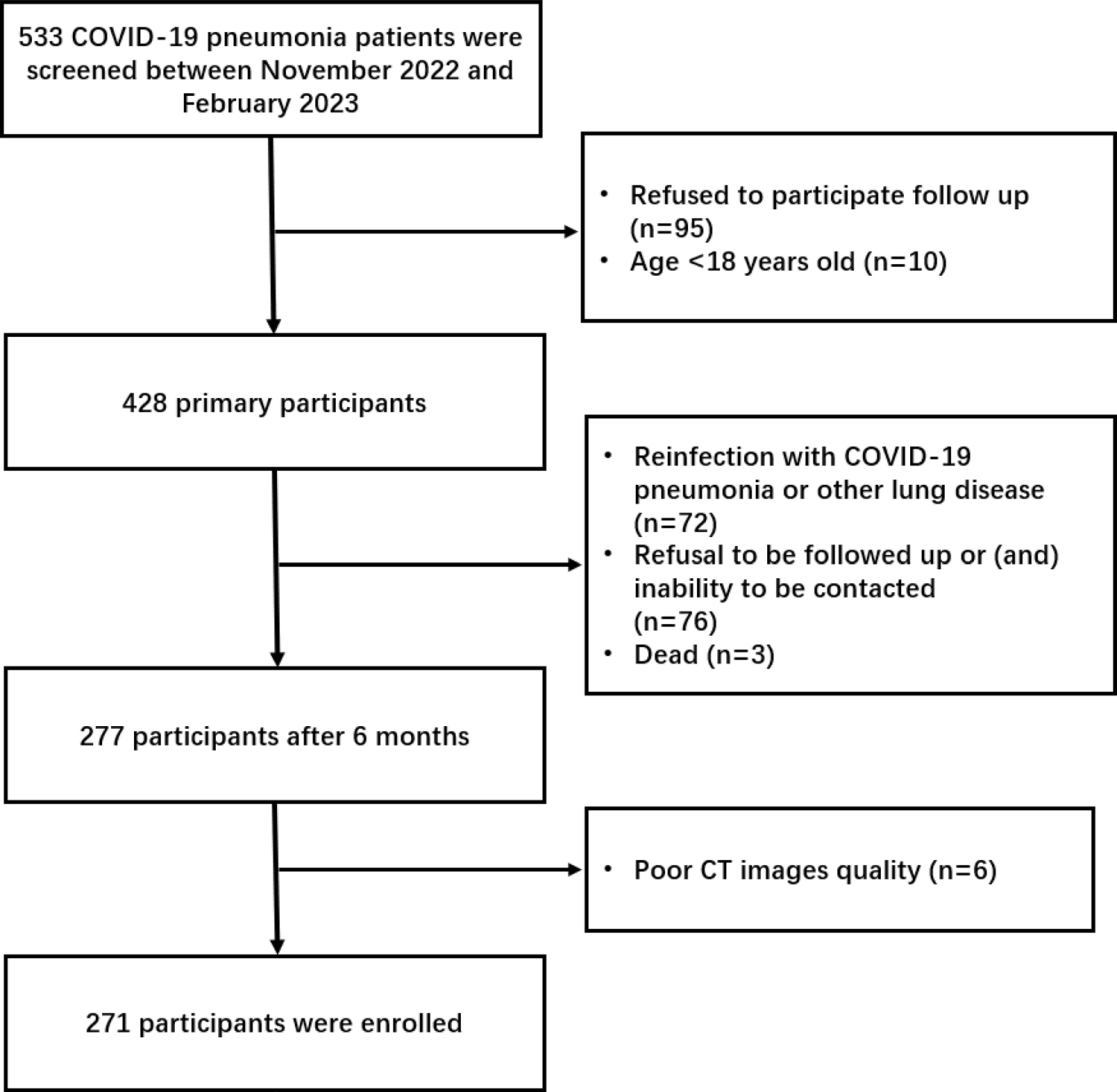
Participant study flowchurt diagram. COVID-19 = coronavirus disease 2019.

We prospectively enrolled 271 patients with COVID-19 pneumonia who had been admitted to either the 305 Hospital of PLA or the 7th Medical Center of Chinese PLA General Hospital between November 29, 2022 and February 10, 2023, when COVID-19 infections increased following gradual deregulation in China. All participants were diagnosis of COVID-19 pneumonia confirmed by means of a SARS-CoV-2 positive polymerase chain reaction test via nasopharyngeal swabs and underwent an initial chest CT to confirm the pneumonia. All positive specimens sent to the Centers for Disease Control and Prevention (CDC) were confirmed to be Omicron BF.7 and BF.5.2. The exclusion criteria included: age less than 18 years, reinfection with COVID-19 pneumonia or other lung disease during the 6-month follow-up period, refusal to be followed up or (and) inability to be contacted, and poor CT images quality (Fig. 1).

### CT protocol

Non-contrast chest scans were obtained with a 64-section multidetector CT scanner (LightSpeed VCT; GE Medical Systems) or a 128-section multidetector CT scanner (Brilliance iCT; Philips Healthcare), with participants in the supine position during a breath hold following full inspiration. The scanning parameters were 120 kV and adaptive tube current, with the smallest field of view possible according to the body habitus. Axial reconstructions were performed with a section thickness of 1 or 1.25 mm using a bone filter. All 271 patients underwent initial CT scans and 6-month follow-up CT scans using the same parameters.

### Image interpretation

All CT images were reviewed in random order by three radiologists (Y.Z, L.L, W.L, with 18, 17 and 10 years of experience in thoracic radiology, respectively), who were blinded to the baseline and clinical information of the participants. The readers independently assessed the CT features using axial images. Multiplane reconstruction is used to resolve any interpretive doubts. Images were interpreted at a window of 1000 to 2000 Hounsfield units and a level of − 700 to − 500 Hounsfield units, respectively, to assess the lung parenchyma.

CT features were described according to the Fleischner Society glossary^14^ as follows: ground-glass opacities (GGO), consolidation, reticulation, linear atelectasis, traction bronchiectasis, parenchymal bands, honeycombing, acute respiratory distress syndrome (ARDS) pattern, crazy paving pattern, organizing pneumonia and pleural effusion. The CT evidence of pulmonary fibrotic-like changes was defined as presence of linear atelectasis, traction bronchiectasis, parenchymal bands and honeycombing^7,14,15^.

The chest CT score is calculated per each of the five lung lobes based on the extent of parenchymal involvement^16^, as follows: (0) no involvement; (1) < 5% involvement; (2) 5–25% involvement; (3) 26–50% involvement; (4) 51–75% involvement; and (5) > 75% involvement. The resulting total CT score is the sum of each individual lobar score and ranges from 0 to 25.

### Statistical analysis

The statistical analyses were performed using the software SPSS 25.0 (IBM Corp., Armonk, NY, USA). Continuous variables are expressed as medians with interquartile ranges (IQRs) or means ± standard deviations (SDs). Categorical variables are reported as numbers and percentages. Continuous variables with normally and nonnormally distributed data were assessed using the two-sample t test or Mann–Whitney U test, respectively. Univariable and multivariable logistic regression analyses were performed to identify the predictive factors of abnormalities or fibrotic-like changes. Statistically significant difference was considered at P < 0.05 (two tailed). Bonferroni correction was used as appropriate.

## Result

### Participant characteristics

A total of 271 participants (mean ± SD, 61 years ± 12) were assessed, and 113 participants were women (41.7%). The baseline and clinical characteristics are summarized in Table 1. Of the 271 participants, the median body mass index was 21.8 kg/m^2^ (IQR, 17.1–29.1), and 80 (29.5%) were smokers. 148 participants (54.6%) had different types of comorbidities and common comorbidities included hypertension (82 participants, 30.3%), type II diabetes mellitus (80 participants, 29.5%), ischemic heart disease (61 participants, 22.5%), chronic obstructive pulmonary disease (18 participants, 6.6%) and previous venous thromboembolism (10 participants, 3.7%). The median hospital stay was 12 days (IQR, 4–20 days), with 68 participants (25.1%) requiring the highest level of ventilatory support in the form of invasive ventilation or noninvasive positive pressure ventilation. Participants are treated with medications mainly including paxlovid (183 participants, 67.5%), azvudine (60 participants, 22.1%) and glucocorticoid (69 participants, 25.5%).

**Table 1:** Comparison of baseline and clinical characteristics between participants with normal and abnormal CT in the lung at 6-month follow-up.

Baseline Characteristics	All participants (n = 271)	Normal CT (n = 173)	Abnormal CT (n = 98)	P Value
Age, year*	61 ± 12	58 ± 11	65 ± 12	< .001
Sex, female	113 (41.7)	67 (38.7)	46 (46.9)	.27
Body mass index, kg/m^2^†	21.8 (17.1–29.1)	20.7 (16.4–27.3)	22.9 (17.7–30.3)	.65
Smokers	80 (29.5)	42 (24.3)	38 (38.8)	.04
Heart rate (beats/min)*	87 ± 15	83 ± 14	92 ± 16	.02
Respiratory rate*	21 ± 8	20 ± 7	24 ± 9	.03
SaO2 on room air, %†	95 (85–98)	96 (88–99)	92 (80–98)	.001
Comorbidities	148 (54.6)	92 (53.8)	56 (57.1)	.61
Hypertension	82 (30.3)	52 (30.0)	30 (30.6)	.92
T2DM	80 (29.5)	49 (28.3)	31 (31.6)	.62
IHD	61 (22.5)	42 (24.2)	19 (19.3)	.47
COPD	18 (6.6)	10 (5.8)	8 (8.1)	.02
Previous VTE	10 (3.7)	7 (4.1)	3 (3.1)	.09
Length of hospital stay, day†	12 (4–20)	11 (4–14)	16 (10–27)	< .001
Ventilatory support	68 (25.1)	31 (17.9)	37 (37.6)	< .001
Noninvasive ventilation	51 (18.8)	29 (16.8)	22 (22.4)	.09
Invasive ventilation	17 (6.3)	2 (1.6)	15 (15.3)	< .001
Medication				
Paxlovid	183 (67.5)	147 (85.0)	36 (36.7)	< .001
Azvudine	60 (22.1)	41 (23.7)	19 (19.4)	.71
Glucocorticoid	69 (25.5)	48 (27.7)	21 (21.4)	.19

Except where indicated, data are numbers of participants, with percentages in parentheses.

*Data are means ± SDs.

^†^Data are medians, with IQR in parentheses.

*SaO2* Oxygen saturation, *IHD* ischemic heart disease, *T2DM* type 2 diabetes mellitus, *COPD* chronic obstructive pulmonary disease, *VTE* venous thromboembolism.

Compared of baseline and clinical characteristics, age (mean, 58 years ± 11 vs 65 years ± 12, P < 0.001), smoker (42 participants [24.3%] vs 38 participants [38.8%], P = 0.04), heart rate (mean, 83 times per minute ± 14 vs 92 times per minute ± 16, P = 0.02), respiratory rate (mean, 20 times per minute ± 7 vs 24 times per minute ± 9, P = 0.03), oxygen saturation on room air (SaO2, 96%, IQR, 88–99% vs 92%, IQR, 80–98%, P = 0.001), chronic obstructive pulmonary disease (COPD, 10 participants [5.8%] vs 8 participants [8.1%], P = 0.02), length of hospital stay (11 days, IQR, 4–14 days vs 16 days, IQR, 10–27 days, P < 0.001), invasive ventilation (2 participants [1.6%] vs 15 participants [15.3%], P < 0.001) and using paxlovid (147 participants [85.0%] vs 36 participants [36.7%], P < 0.001) demonstrated a statistically significant difference between participants with normal and abnormal chest CT at 6-month follow-up.

### Comparison of CT findings

**Figure 2:**
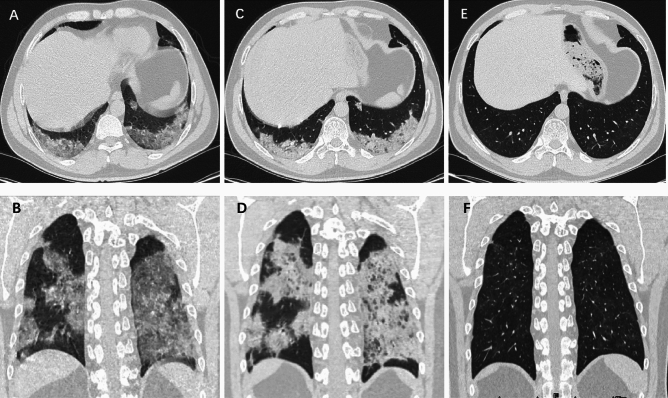
Serial chest CT scans in a 45-year-old man with severe coronavirus disease 2019 pneumonia. (**A**, **B**) Initial CT scans obtained on day 5 after the onset of symptoms showed extensive ground-glass opacities (GGO) with some areas of consolidation bilaterally. (**C**, **D**) CT scans obtained on day 9 showed extensive consolidation with few GGOs bilaterally. (**E**, **F**) CT scans obtained on day 179 showed almost absorption of the abnormalities with mild GGOs and interstitial thickening remaining.

**Figure 3:**
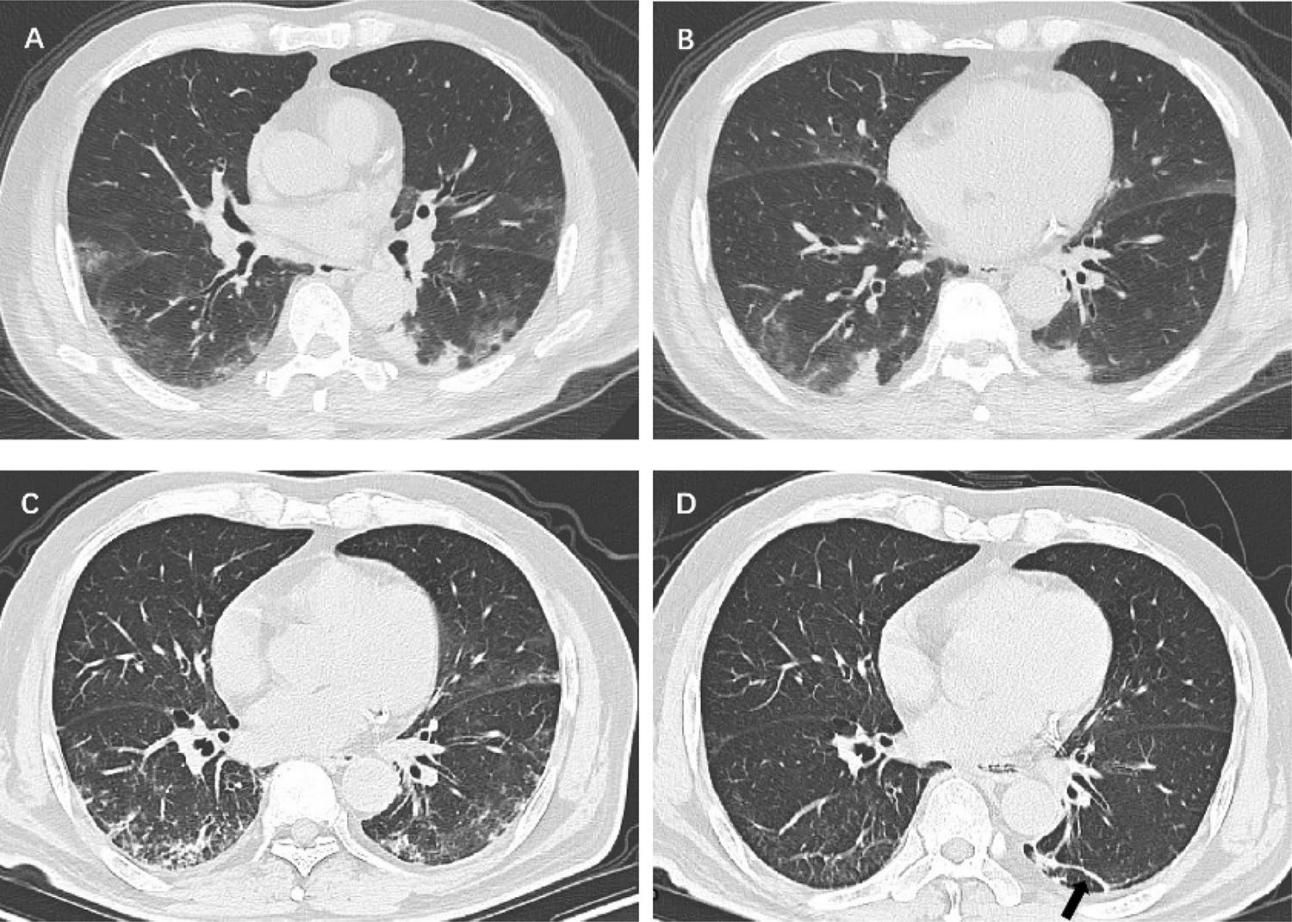
Serial chest CT scans in a 61-year-old man with coronavirus disease 2019 pneumonia. (**A**, **B**) Initial CT scans obtained on day 4 after the onset of symptoms showed multiple ground-glass opacities and consolidation bilaterally. (**C**) CT scans obtained on day 22 showed moderate consolidation and reticulation in the lower lung lobes bilaterally. (**D**) CT scans obtained on day 191 showed obviously absorption of the abnormalities with subtle reticulation and linear atelectasis (arrow) in the lower lung lobes.

**Figure 4:**
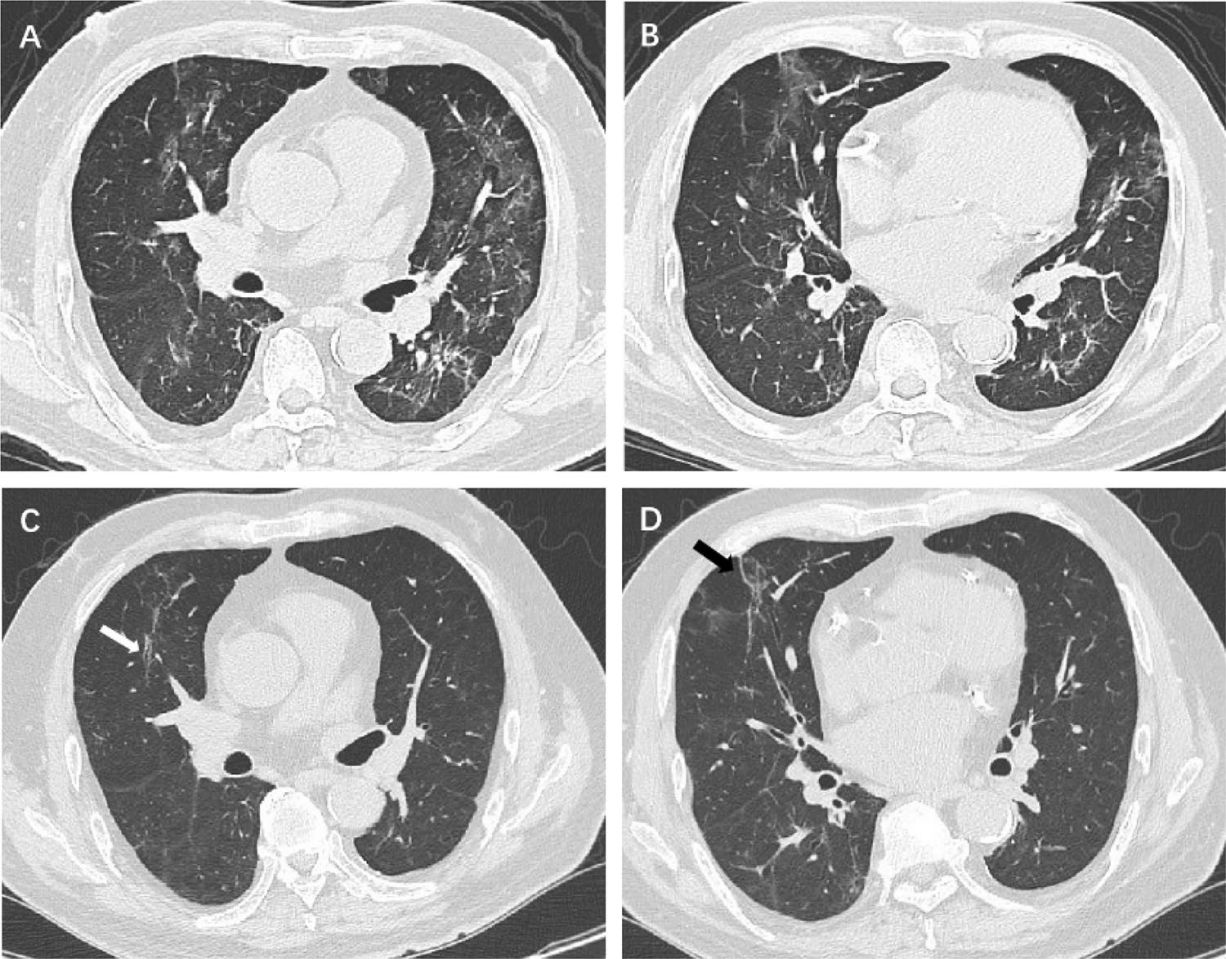
Serial chest CT scans in a 60-year-old man with coronavirus disease 2019 pneumonia. (**A**, **B**) Initial CT scans obtained on day 8 after the onset of symptoms showed multiple ground-glass opacities and interstitial thickening bilaterally. (**C**, **D**) CT scans obtained on day 180 showed traction bronchiectasis (white arrow) and interlobar pleural traction (black arrow) in the upper lobe of right lung.

**Figure 5:**
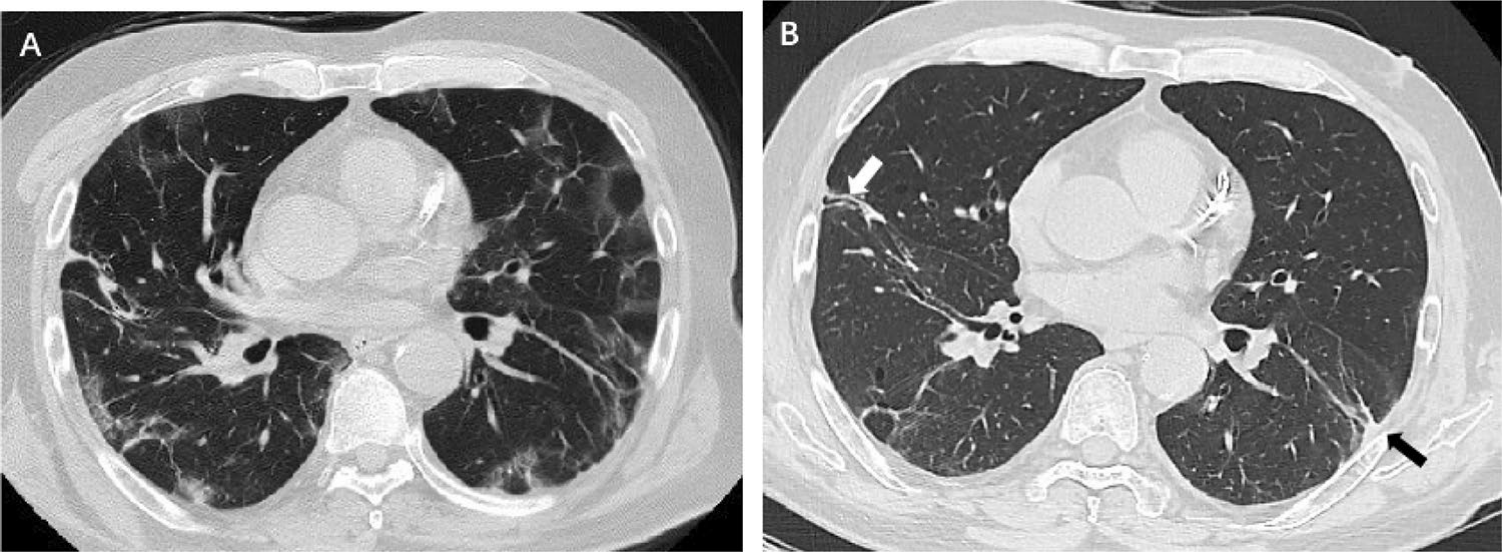
Serial chest CT scans in a 54-year-old man with coronavirus disease 2019 pneumonia. (**A**) Initial CT scans obtained on day 9 after the onset of symptoms showed multiple ground-glass opacities and interstitial thickening bilaterally. (**B**)CT scans obtained on day 169 showed traction bronchiectasis (white arrow) and parenchymal bands (black arrow) in the lower lung lobes.

All participants underwent a 6-month follow-up chest CT at a median of 177 days (IQR, 155–203 days) after hospital admission and pulmonary residual abnormalities were found in 98 participants (36.2%). Compared to the initial CT (Table 2), participants with GGO decreased from 270 (99.6%) to 66 (24.4%) and consolidation decreased from 111 (41.0%) to 20 (7.4%) (Fig. 2). Meanwhile, participants with reticulation increased from 19 (7.0%) to 57 (21.0%). The ARDS pattern in three participants (1.1%) and crazy paving pattern in two participants (0.7%) at initial CT had disappeared at 6-month follow-up CT. Participants with organizing pneumonia pattern increased from four (1.5%) to seven (2.6%). Among CT evidence of fibrotic-like changes, participants with linear atelectasis increased from four (1.5%) to seven (2.6%) (Fig. 3), participants with bronchiectasis and parenchymal bands increased from six (2.2%) to 31 (11.4%) (Fig. 4) and 14 (5.2%) (Fig. 5) respectively. There was no change in the three participants (1.1%) with honeycombing. In summary, 39 participants (14.4%) demonstrated new suspicious fibrotic-like changes at 6-month follow-up CT.

**Table 2:** Comparison of CT Findings in the lung between initial and 6-month follow-up CT.

CT feature	Initial CT (n = 271)	6-month CT (n = 271)	P value
Any abnormality	271 (100)	98 (36.2)	< .001
GGO	270 (99.6)	66 (24.4)	< .001
Consolidation	111(41.0)	20 (7.4)	< .001
Reticulation	19 (7.0)	57 (21.0)	< .001
Fibrotic-like changes	16 (5.9)	55 (20.3)	< .001
Linear atelectasis	4 (1.5)	7 (2.6)	0.08
Traction bronchiectasis	6 (2.2)	31 (11.4)	< .001
Parenchymal bands	6 (2.2)	14 (5.2)	0.02
Honeycombing	3 (1.1)	3 (1.1)	0.99
ARDS pattern	3 (1.1)	0 (0)	0.01
Crazy paving pattern	2 (0.7)	0 (0)	0.02
Organizing pneumonia	4 (1.5)	7 (2.6)	0.08
Pleural effusion	11 (4.1)	3 (1.1)	0.01

Data are numbers of participants, with percentages in parentheses.

*GGO* ground-glass opacity. *ARDS* acute respiratory distress syndrome.

### Comparison of chest CT scores

In the Chest CT scores (Table 3), a significantly decrease was found for any abnormality (P < 0.001), GGO (P < 0.001), and consolidation (P < 0.001), whereas a significantly increase for fibrotic-like abnormalities (P < 0.001) compared with the initial CT scans. Meanwhile, reticulation showed insignificantly change between two CT scans (P = 0.33).

**Table 3:** Comparison of Chest CT Scores between initial and 6-month Follow-up CT.

Chest CT Scores	Initial CT (n = 271)	6-month CT (n = 271)	P value
Any abnormality	14 (9)	3 (9)	< .001
GGO	11 (8)	2 (8)	< .001
Consolidation	4 (7)	1 (3)	< .001
Reticulation	4 (6)	3 (7)	.33
Fibrotic-like	0 (1)	0 (4)	< .001

Data are presented as medians (interquartile ranges).

*GGO* ground-glass opacity.

### Factors associated with pulmonary residual abnormalities

In the univariate analysis, paxlovid (odd ratio [OR]: 0.08; 95% CI 0.03, 0.21; P < 0.001), invasive ventilation (OR 9.3; 95% CI 2.8, 29; P < 0.001), age > 60 years (OR 6.5; 95% CI 2.7, 17; P < 0.001), SaO2 less than 93% at admission (OR 4.5; 95% CI 1.4, 14; P < 0.001), hospitalization more than 15 days (OR 3.8; 95% CI 1.3, 11; P = 0.002), and respiratory rate more than 23 times per minute at admission (OR 3.3; 95% CI 1.3, 8.7; P = 0.004) were associated with pulmonary residual abnormalities at 6-month follow-up CT. In the multivariate analysis, the predictive factors were invasive ventilation (OR 13.6; 95% CI 1.9, 45; P < 0.001), age > 60 years (OR 9.1; 95% CI 2.3, 39; P = 0.01), paxlovid (OR 0.11; 95% CI 0.04, 0.48; P = 0.01), hospitalization more than 15 days (OR 6.1; 95% CI 1.2, 26; P = 0.002), heart rate greater than 100 times per minute (OR 5.9; 95% CI 1.1, 27; P = 0.03), and SaO2 less than 93% at admission (OR 5.6; 95% CI 1.4, 13; P = 0.02) (Table 4).

**Table 4:** Univariable and multivariable analysis of pulmonary residual abnormalities at 6-month follow-up CT.

Predictors	Univariable Analysis (n = 271)			Multivariable Analysis (n = 271)		
	OR	95% CI	P Value	OR	95% CI	P Value
Age > 60 years	6.5	2.7–17	< .001	9.1	2.3–39	.01
Heart rate > 100 times/min	2.3	1.1–6.5-	.063	5.9	1.1–27	.03
SaO2 < 93%	4.5	1.4–14	< .001	5.6	1.4–13	.02
Hospitalization > 15days	3.8	1.3–11	.002	6.1	1.2–26	.02
Invasive ventilation	9.3	2.8–29	< .001	13.6	1.9–45	< .001
Paxlovid	0.08	0.03-0.21	< .001	0.11	0.04-0.48	.01

### Factors associated with pulmonary fibrotic-like changes

In the univariate analysis, paxlovid (OR 0.11; 95% CI 0.04, 0.32; P < 0.001), invasive ventilation (OR 8.8; 95% CI 2.1, 26; P < 0.001), smoker (OR 7.4; 95% CI 3.0, 16; P < 0.001), SaO2 less than 93% at admission (OR 4.5; 95% CI 1.2, 16; P = 0.002) and age > 60 years (OR 4.2; 95% CI 1.3, 11; P = 0.002) were associated with pulmonary fibrotic-like changes at 6-month follow-up CT. In the multivariate analysis, the predictive factors were invasive ventilation (OR 10.3; 95% CI 2.9, 33; P = 0.002), smoker (OR 9.9; 95% CI 2.4, 31; P = 0.01), paxlovid (OR 0.1; 95% CI 0.03, 0.48; P = 0.01), SaO2 less than 93% at admission (OR 7.8; 95% CI 1.5, 19; P = 0.02), age > 60 years (OR 6.1; 95% CI 2.3, 22; P = 0.03) and heart rate greater than 100 times per minute (OR 4.9; 95% CI 1.7, 11; P = 0.04) (Table 5).

**Table 5:** Univariable and multivariable analysis of pulmonary fibrotic-like changes at 6-month follow-up CT.

Predictors	Univariable Analysis (n = 98)			Multivariable Analysis (n = 98)		
	OR	95% CI	P Value	OR	95% CI	P Value
Age > 60 years	4.2	1.3–11	.002	6.1	2.3–22	.03
Smoker	7.4	3.0–16	< .001	9.9	2.4–31	.01
Heart rate > 100 times/min	2.3	0.9–7.7	.074	4.9	1.7–11	.04
SaO2 < 93%	4.5	1.2–16	.002	7.8	1.5–19	.02
Invasive ventilation	8.8	2.1–26	< .001	10.3	2.9–33	.002
Paxlovid	0.11	0.04-0.32	< .001	0.1	0.03-0.48	.01

## Discussion

We prospectively followed COVID-19 pneumonia patients discharged from hospitals in China during the turn of 2022–2023 with chest CT to better understand the radiologic change with time. In several follow-up studies of COVID-19 pneumonia patients infected in 2020, the results indicated that 48–78% of the patients still had pulmonary abnormalities at 6-month follow-up^5,17–19^. A study of COVID-19 patients infected from February to May 2021 found that at least 66% of patients still had pulmonary abnormalities at 6-month follow-up CT, but the mean age of the patients were 82.3 years ± 7.1 (SD)^20^. With the worldwide phasing out of universal SARS-CoV-2 polymerase chain reaction test for the population until the increase of COVID-19 cases in China at the end of 2022, no new follow-up studies have been reported. In our study, 98 of 271 participants (36.2%) had radiographic abnormalities at the 6-month follow-up, with the most common findings being GGO (66 of 271 participants, 24.4%), and reticulation (57 of 271 participants, 21%). Our study showed the proportion of patients having residual pulmonary abnormalities in 6-month CT follow-up decreases after 2–3 years of genetic mutation of SARS-CoV-2. However, the most common pulmonary abnormalities were also GGO and reticulation, which did not differ from several of the studies mentioned above.

Pulmonary fibrosis is one of the most important concerns in the long-term follow-up of CT in COVID-19. Most studies judged pulmonary fibrosis based on the interpretation of the Fleischner Society glossary for CT features. Linear atelectasis, traction bronchiectasis, parenchymal banding and honeycombing are the most common features used to evaluate pulmonary fibrosis. Several studies have reported pulmonary fibrosis and(or) fibrotic-like changes in 35–56% of COVID-19 patients in 6-month CT follow-up^7,20,21^. In our study, 39 of 271 participants (14.4%) had fibrotic-like changes at the 6-month follow-up CT. The presence of fibrotic-like changes was reduced in our study compared to studies mentioned above. However, the selection and recognition of the relevant CT features differed somewhat in diverse studies. Most of the fibrotic-like changes in COVID-19 patients would disappear up to 1 year post hospital discharge^21–23^. It has been reported that in survivors of the preceding SARS pandemic, fibrotic-like changes present at 6 months may still improve at 84 months of follow-up, such as traction bronchiectasis^24^. Actually, periods of fibroproliferation of variable severity are part of the natural history of diffuse alveolar damage^25,26^. Most fibrotic-like changes are reversible, so it should be cautious to describe them as fibrotic changes or pulmonary fibrosis. The exact proportion of patients with long-term, irreversible fibrotic changes is unknown, but appears to be low, and the proportion of progressive pulmonary fibrosis is expected to be even lower, thus long-term follow-up studies are necessary^27^.

In both multivariable analyses of residual pulmonary abnormalities and pulmonary fibrotic-like changes, invasive ventilation ranked first among the risk factors. First, these patients were admitted to the intensive care unit, reflecting the severity of the disease and the slow recovery. In addition, some studies have confirmed that medically induced injury from invasive mechanical ventilation is probably an important cause of pulmonary fibrosis^26,28^. Paxlovid is another important independent prognostic factor, and it has been proven to be a very effective antiviral drug against SARS-CoV-2^29–31^. Paxlovid, azvudine and glucocorticoid were the three most widely used drugs in the medication of the COVID-19 pneumonia in Beijing epidemic. Azvudine is another effective antiviral drug, which can increase the rate of nucleic acid negative conversion and early hospital discharge^32,33^. Glucocorticoid is effective in reducing mortality in severe COVID-19 patients^34,35^. However, in this study, using of Azvudine or glucocorticoid at the time of infection was not found to be effective in improving pulmonary residual abnormalities and fibrotic-like changes at 6-month follow-up. A reason for this may be that as an antiviral drug, the effect of Azvudine in inhibiting SARS-CoV-2 is weaker than that of Paxlovid, which was confirmed in a recent study^36^. Glucocorticoid acts to reduce the inflammatory response in the acute phase^37^, and may not relieve lung lesions in the chronic phase.

The long-term follow-up of COVID-19 is necessary but will inevitably increase considerable amount of work. Artificial intelligence (AI) may be helpful in detecting lung abnormalities and improving efficiency. AI models help in visualization and measurement of COVID-19 specific lesions in the lungs of infected patients, potentially facilitating timely, patient-specific medical interventions^38^. Moreover, AI has a good correlation with radiologists in detecting lung lesions, but more efficiently^39^.

This study has some limitations. First, this study did not correlate the performance of CT with clinical symptoms, laboratory examinations, and especially abnormalities in lung function, which was greatly restricted during the epidemic due to infectious disease control and prevention, and therefore could not be compared with lung function results at follow-up. However, changes in lung function play an important role in evaluating the regression of COVID-19 pneumonia and interstitial fibrosis in patients^40^. So, lack of relevant data makes a limitation of our study. Second, at the beginning of the COVID-19 epidemic in Beijing, due to the lack of paxlovid, some patients did not start paxlovid until the 2nd–4th day after admission to the hospital. Gradually, patients were able to ensure that standardized paxlovid therapy as soon as they were admitted to the hospital. This difference may have had some impact on the results. However, even so, the results suggest that paxlovid may has a promising effect on the long-term regression of the COVID-19 pneumonia.

In conclusion, this prospective study showed that in the COVID-19 pandemic of China during the turn of 2022–2023, a considerable proportion of the patients with pulmonary residual abnormalities and fibrotic-like changes were found at the 6-month follow-up CT. Furthermore, it was observed that antivirals against SARS-CoV-2 like paxlovid may be beneficial for long-term regression of COVID-19. While it is commonly assumed that the genetic mutations of SARS-CoV-2 have led to a significant decrease in its pathogenicity, our study indicates the importance of long-term follow-up for patients with COVID-19 pneumonia.

## Data Availability

The datasets used and/or analysed during the current study available from the corresponding author on reasonable request.

## Abbreviations

COVID-19: Coronavirus disease 2019
SARS-CoV-2: Severe acute respiratory syndrome coronavirus type 2
GGO: Ground-glass opacity
ARDS: Acute respiratory distress syndrome
